# Growth Hormone Receptor Controls Adipogenic Differentiation of Chicken Bone Marrow Mesenchymal Stem Cells by Affecting Mitochondrial Biogenesis and Mitochondrial Function

**DOI:** 10.3389/fcell.2022.827623

**Published:** 2022-03-08

**Authors:** Changbin Zhao, Bowen Hu, Zhiying Liao, Haohui Wei, Yongxia Zhao, Jinping Liang, Wen Luo, Qinghua Nie, Qingbin Luo, Dexiang Zhang, Xiquan Zhang, Hongmei Li

**Affiliations:** ^1^ Lingnan Guangdong Laboratory of Modern Agriculture, College of Animal Science, South China Agricultural University, Guangzhou, China; ^2^ State Key Laboratory for Conservation and Utilization of Subtropical Agro-bioresources, College of Animal Science, South China Agricultural University, Guangzhou, China; ^3^ Guangdong Provincial Key Lab of Agro-Animal Genomics, Ministry of Agriculture, Guangzhou, China; ^4^ Molecular Breeding and Key Lab of Chicken Genetics, Breeding and Reproduction, Ministry of Agriculture, Guangzhou, China

**Keywords:** GHR, bone mesenchymal stem cells, mitochondrial biogenesis, mitochondrial function, adipogenic differentiation, sex-linked dwarf chickens

## Abstract

Growth hormone receptor (GHR) can activate several signaling pathways after binding to growth hormone (GH) to regulate cell growth and development. Sex-linked dwarf (SLD) chickens, normal protein functions are prevented because of exon mutations in the *GHR* gene, have more severe fat deposition. However, the specific molecular mechanisms responsible for this phenotype remains unclear. We therefore investigated the effect of the *GHR* gene on adipogenic differentiation of chicken bone marrow mesenchymal stem cells (BMSCs). We found that bone marrow fat deposition was more severe in SLD chickens compared to normal chickens, and the expression of genes related to adipogenic differentiation was enhanced in SLD chicken BMSCs. We also detected enhanced mitochondrial function of BMSCs in SLD chickens. *In vitro*, overexpression of *GHR* in chicken BMSCs increased mitochondrial membrane potential but decreased reactive oxygen and ATP contents, oxidative phosphorylation complex enzyme activity, and mitochondrial number. Expression of genes associated with mitochondrial biogenesis and function was repressed during adipogenic differentiation in chicken BMSCs, the adipogenic differentiation capacity of chicken BMSCs was also repressed. With knockdown of *GHR*, opposite results were observed. We concluded that *GHR* inhibited adipogenic differentiation of chicken BMSCs by suppressing mitochondrial biogenesis and mitochondrial function.

## Introduction

Growth hormone (GH), a peptide hormone regulated by the hypothalamus and secreted by the pituitary gland, binds to cell surface growth hormone receptors to regulate metabolic processes and growth and development (Hu et al., 2021). Growth hormone receptor (GHR), a member of the type I cytokine receptor family, is the key receptor transmembrane protein in the GH–GHR axis. The GHR protein has three primary domains: extracellular, single-pass transmembrane, and cytoplasmic intracellular (Dehkhoda et al., 2018). In sex-linked dwarf (SLD) chickens, normal protein functions are prevented because of exon mutations in the *GHR* gene, compared with normal chickens (Burnside et al., 1991). Sex-linked dwarf chickens are short, weigh only 60–70% of normal chickens, and have higher feed utilization than normal but also more severe fat deposition (Guillaume, 1976). The molecular mechanisms responsible for the SLD phenotype are not clearly understood.

GH can regulate mitochondrial respiration by binding to the Box1 region of the *GHR* gene (Perret-Vivancos et al., 2006). In *GHR* knockout (GHRKO) mice, mitochondrial function and antioxidant capacity increase (Brown-Borg et al., 2009). Mitochondrial biogenesis maintains mitochondrial homeostasis and function by producing new mitochondria (Ploumi et al., 2017), and in GHRKO mice, key regulators of mitochondrial biogenesis increase in liver, kidney, and skeletal muscle (Gesing et al., 2011a; Gesing et al., 2011b). However, those results conflict with those of other reports. Mitochondrial function is severely impaired in osteoblasts and fibroblasts of GHRKO mice (Liu et al., 2019), and deficiency of *GHR* function impairs mitochondrial function in chicken skeletal muscle and DF-1 cells (Hu et al., 2019). Such conflicting results suggest that the effects of *GHR* on mitochondria vary by species and tissue.

Bone mesenchymal stem cells (BMSCs), also known as bone marrow-derived mesenchymal stem cells, are multipotent stromal cells with self-renewing ability and multilineage differentiation (Muruganandan et al., 2009). They can differentiate into many different types of cells, including adipocytes, osteoblasts, chondrocytes, myocytes, and neurons (Attia and Mashal, 2020). Most current research on BMSCs focuses on both adipogenesis and osteogenesis. Adipogenic and osteogenic differentiation of BMSCs is regulated by multiple signaling and transcription factors (James, 2013). In general, adipogenic differentiation and osteogenic differentiation in BMSCs are mutually exclusive, with stimulation of osteogenesis suppressing adipogenesis and vice versa (Yang et al., 2014). When expression of peroxisome proliferator-activated receptor-γ (PPARγ) is suppressed, the ability of BMSCs to differentiate toward adipogenesis diminishes, whereas the ability to differentiate toward osteogenesis increases (Li et al., 2017). In GHRKO mice, expression of genes associated with adipogenic differentiation increases in mesenchymal stem cells (MSCs), and the ability to differentiate toward adipogenesis also increases (Olarescu et al., 2015). However, specific mechanisms of the GH–GHR axis and adipogenic differentiation of BMSCs are unclear.

During proliferation of MSCs, the primary metabolic mode is glycolysis, whereas during their differentiation, the primary mode shifts from glycolysis to mitochondrial-based oxidative phosphorylation (Hsu et al., 2016). Reactive oxygen species (ROS) produced by mitochondrial complex III are required to initiate adipocyte differentiation (Tormos et al., 2011). A key regulator of mitochondrial biogenesis, *PGC1α*, promotes adipogenic differentiation and inhibits osteogenic differentiation in immortalized human MSCs (Huang et al., 2011). Mitochondria are highly dynamic organelles that can be rapidly restructured to meet metabolic demands in a timely manner (Wang and Hai, 2015). Thus, mitochondria are essential in adipogenic differentiation of BMSCs.

In SLD chickens, liver mitochondrial function declines compared with that in normal chickens (Li et al., 2019). Therefore, SLD and normal chickens were compared in this study, and overexpression and knockdown of *GHR* in BMSCs were used to determine the effects of *GHR* on mitochondrial function and adipogenic differentiation of chicken BMSCs.

## Materials and Methods

### Ethics Statement

All animal experiments were performed according to the protocols approved by the South China Agriculture University Institutional Animal Care and Use Committee (approval number SCAU#0017). All animal procedures followed the regulations and guidelines established by this committee and minimized the suffering of animals.

### Chickens

For *in vivo* experiments, the Guangdong Wenshi Southern Poultry Breeding Co., Ltd. (Guangzhou, China), provided 21-day-old yellow-feather chickens. To isolate bone BMSCs, the Yuhe Agriculture and Animal Husbandry Co., Ltd. (Guangzhou, China), provided 3-day-old chickens.

The 21-day-old yellow-feather chickens included 15 SLD chickens and 15 normal chickens. To explore molecular mechanisms of *GHR in vivo* and determine the cause of fatty deposits in SLD chickens, mitochondrial function, mitochondrial biogenesis, and adipogenic differentiation in chicken BMSCs were examined in those chickens. The 3-day-old normal chickens were only used to isolate BMSCs, and the BMSCs were used to study *GHR* effects on mitochondria and adipogenic differentiation *in vitro*.

### Paraffin Sections and Hematoxylin and Eosin Staining.

The epiphyses of thighbone from the 21-day-old SLD and normal chickens were fixed with 10% neutral formalin for 5 days, then immersed in 10% hydrochloric acid formaldehyde solution (10 ml hydrochloric acid +10 ml formaldehyde +80 ml water) for 1 h, rinsed in running water for 2 h. The samples were dehydrated in gradient alcohol and transparent with xylene after decalcification, details as follow: 75% alcohol for 2 h; 85% alcohol for 2 h; 90% alcohol for 1.5 h; 95% alcohol for 2 h; anhydrous ethanol I for 2 h; anhydrous ethanol II for 2 h; alcohol benzene for 40 min; xylene I for 40 min; xylene II for 40 min; 65°C melted paraffin I for 0.5 h at 65°C; melted paraffin II for 1 h; 65° melted paraffin III for 2 h 45 min. The wax-soaked tissues were embedded in the embedding machine, a paraffin sectioning machine cut 7 to 10-μm-thick sections. After that, hematoxylin and eosin staining was routinely performed. The first step is dewaxing, the steps are as follows: xylene I for 20 min; xylene II for 20 min; 100% ethanol I for 5 min; 100% ethanol II for 5 min; 75% ethanol for 5 min; rinsing with tap water. Then stain sections with hematoxylin solution for 3–5 min, rinse with tap water. Treat the section with hematoxylin differentiation solution, rinse with tap water. Treat the section with Hematoxylin Scott Tap Bluing, rinse with tap water. The sections were then immersed in 85% ethanol for 5 min, 95% ethanol for 5 min, and stained with eosin dye for 5 min. Sections were dehydrated with ethanol and xylene in turn and finally sealed with neutral gum. All the sections were analyzed by microscope (Leica DMi8, Wetzlar, Germany).

## Frozen Sections and Oil Red Staining

Epiphyseal parts of femurs of the 21-day-old SLD and normal chickens were cut off and soaked in 4% paraformaldehyde for 48 h and then switched to decalcification solution (Servicebio, Wuhan, China) for 30 days, with the solution changed every 2 days. After decalcification, tissues were placed in a 15% sucrose solution in a refrigerator at 4°C to dehydrate and sink and then were transferred to a 30% sucrose solution at 4°C to dehydrate and sink. Dehydrated tissue was placed cut side up on a sample tray and surrounded by drops of OCT embedding agent (Servicebio). The tray was placed on the quick-freeze table of a frozen sectioning machine, and the samples were sectioned after the OCT whitened and hardened. Section thickness was 8–10 μm. Then stain sections with Oil Red solution (Servicebio) for 8–10 min in the dark, and cover it with lid during dyeing. Take out the slices, stay for 3 s and then immerse them in two cups of 60% isopropanol for differentiation in turn, 3 and 5 s respectively. The slices were immersed to two cups of pure water in turn for 10 s each. Take out the slices, immerse in hematoxylin for 3–5 min after 3s, and then rinse in three cups of pure water for 5, 10, and 30 s in turn. Treat it with differentiation solution (60% alcohol as solvent) for 2–8 s, two cups of distilled water for 10 s each, and Scott Tap Bluing for 1 s. Then lightly dip the slices in two cups of tap water for 5 and 10 s in turn and finally seal the slices with glycerin gelatin. All the sections were analyzed by microscope (Leica DMi8).

### Detection of Triglyceride.

Triglyceride was measured using a Triglyceride Assay Kit (Nanjing Jiancheng, Nanjing, China) according to the manufacturer’s protocol. Triglyceride was measured at 510 nm, and absorbance was determined using a Fluorescence/Multi-Detection Microplate Reader (Bio-Tek, Winooski, United States) according to the manufacturer’s protocol. Data were normalized to the control group and expressed as a percentage of the control.

### Reverse-Transcription Quantitative PCR

RNA was extracted from tissues or cells using RNAiso reagent (Takara, Shiga, Japan) according to the manufacturer’s protocol. Concentration of RNA samples and optical density (OD) value of 260/280 were detected using a Nanodrop 2000c spectrophotometer (Thermo, Waltham, United States). Samples were stored at −80°C for later use. For reverse-transcription quantitative PCR (RT-qPCR), cDNA was synthesized using MonScript™RTIII All-in-One Mix with dsDNase (Monad Co., Ltd., Guangzhou, China). ChamQ Universal SYBR qPCR Master Mix (Vazyme, Guangzhou, China) was used in RT-qPCRs run on a Bio-Rad CFX96 Real-Time Detection instrument (Bio-Rad, Hercules, United States) according to the manufacturer’s protocol. The reaction procedure included initial denaturation at 95°C for 3 min, followed by denaturation at 95°C for 10 s and annealing at 60°C for 30 s, for a total of 40 cycles. At the end of the cycle, the dissolution curve was analyzed, and the detection temperature was 65–95°C. Relative gene expression was measured using RT-qPCR twice for each reaction, and β-actin was used as the control. The primers used in RT-qPCR are listed in Table 1.

**TABLE 1:** Primer sequences in reverse-transcription quantitative PCR.

Gene	Primer sequences (5′–3′)	Temperature (°C)	Size (bp)
*GHR*	F-GCAAGTGCAGGTCACCTGAG	56	153
	R-CCGGACATTCTTTCCAGTCT		
*ND1*	F-ACCCAAGAGCCCATCTACCT	56	154
	R-GTCCGGCGGCATATTCTACA		
*ND2*	F-CCGAGCGATTGAAGCCACTA	56	103
	R-TCATTGTCCGGTGGATCAGG		
*CYTB*	F-CAGCAGACACATCCCTAGCC	56	104
	R-GAAGAATGAGGCGCCGTTTG		
*COX1*	F-ACTACTTACCGACCGCAACC	56	132
	R-CCGAAACCTGGGAGGATGAG		
*COX2*	F-TCGGGGTAAAAACAGACGCA	56	70
	R-ACTCCTGGTCGAGTGGTGAT		
*ATP6*	F-TACAGCCACAATCGCCCTAC	56	123
	R-AGGACGAAGACGTAGGCTTG		
*ATP8*	F-AACCCAAACCCATGATTCTCCA	56	139
	R-AGGTTCAGGGGGTGGGTTTA		
*PGC1α*	F-TCCTTTCCTCAACGCAGGTC	56	153
	R-TCTTGCACGTGAGGGAGAAC		
*NRF1*	F-ACGAGGACTCACCTTCCTCA	56	163
	R-TGTGGTCGCTTCCGTTTCTT		
*TFAM*	F-GACCTCGAAGTGGCTTCAAC	56	144
	R-GAGCAAGCTGAAGGTATGGCT		
*PPARγ*	F-CCAGCGACATCGACCAGTTA	60	275
	R-AGAGCGAAACTGACATCGCT		
*CEBP/α*	F-GACAAGAACAGCAACGAGTACC	60	195
	R-CCTGAAGATGCCCCGCAGAGT		
*CEBP/β*	F-AACCTGTCCACCTCGTCCT	60	241
	R-CCAAGACTTTGTGCTGCGTC		
*β-actin*	F-GATATTGCTGCGCTCGTTG	56	178
	R-TTCAGGGTCAGGATACCTCTTT		

### Extraction of Chicken Bone Mesenchymal Stem Cells and Cell Culture.

Bone mesenchymal stem cells were extracted using the appropriate separation kits (TBD science, Tianjin, China) following the manufacturer’s protocol.

Bone mesenchymal stem cells from 21-day-old SLD and normal chickens were extracted by cell separation kits and cultured *in vitro* to the appropriate density (the first generation). Then, assays were conducted on mitochondrial function and related gene expression and protein levels.

Bone mesenchymal stem cells from 3-day-old normal chickens were extracted by cell separation kits and were cultured *in vitro* and passaged to the third generation. Overexpression and knockdown of *GHR* were to explore the effects of *GHR* on mitochondrial and adipogenic differentiation in chicken BMSCs.

Bone mesenchymal stem cells were cultured in Gibco Dulbecco’s Modified Eagle Medium (DMEM): F-12 (Gibco, Waltham, United States) with 10% fetal bovine serum (FBS) (Gibco) and 1% penicillin/streptomycin (Gibco). All cells were cultured at 37°C in a 5% CO_2_ humified atmosphere.

### Induction of Adipogenic Differentiation

Bone mesenchymal stem cells were seeded into 6-well plates at 1.25 × 10^5^ cells per cm^2^. Bone mesenchymal stem cells were induced with adipogenic medium containing DMEM/F12 (10% FBS), 0.5 mM 3-isobutyl-1-methylxanthine (Sigma-Aldrich, Darmstadt, Germany), 1 μM dexamethasone (Sigma-Aldrich), 10 μg/ml insulin (Sigma-Aldrich), and 200 μM indomethacin (Sigma-Aldrich). The medium was replaced every 2 days for 6 days.

### Plasmid Construction, Small Interfering RNA, and Transfection.

Third generation BMSCs were plated onto 6-well plates, and transfection began when the density reached approximately 80%. After 6 h of transfection, the DMEM/F12 medium was changed to adipogenic induction medium to induce adipogenic differentiation of BMSCs.

GeneCreate (Wuhan, China) synthesized the plasmid pcDNA3.1-*GHR*. Plasmid transfection was performed using Lipofectamine 3,000 reagent (Invitrogen, Waltham, United States) following the manufacturer’s protocol, and nucleic acids were diluted in OPTI-MEM (Gibco). All cells were analyzed 72 h after transfection.

Guangzhou RiboBio (Guangzhou, China) synthesized small interfering RNAs (siRNA) used for *GHR* knockdown. In preliminary experiments, four siRNAs were designed to interfere with *GHR*, and the si-*GHR* with the highest interference efficiency was used. The siRNA sequence is provided in Table 2. The si-*GHR* sequence was transfected in BMSCs to a final concentration of 100 nM using Lipofectamine 3,000 reagent (Invitrogen, United States) according to the manufacturer’s protocol. Cells were analyzed at 72 h after transfection.

**TABLE 2:** Oligonucleotide sequence in this study.

Fragment name	Sequence (5′–3′)
si-*GHR*	CCU​CGA​UUU​GGA​UAC​CAU​A

### Detection of Reactive Oxygen Species

Production of ROS in mitochondria was measured using an ROS assay kit (Beyotime, Shanghai, China) according to the manufacturer’s protocol. Dichlorofluorescein (DCF) fluorescence was determined using a Fluorescence/Multi-Detection Microplate Reader (Bio-Tek). Data were normalized to the control group and are expressed as a percentage of the control.

### Detection of ATP Content

ATP levels were measured using an ATP assay kit (Beyotime) according to the manufacturer’s protocol. A Fluorescence/Multi-Detection Microplate Reader (BioTek) was used to determine ATP levels. Data were normalized to the control group and are expressed as a percentage of the control.

### Detection of Mitochondrial Membrane Potential

Mitochondrial membrane potential (ΔΨm) was measured using a JC-1 kit (Beyotime) according to the manufacturer’s protocol. Mitochondria were fixed with JC-1, and after cells were incubated with JC-1 for 20 min at 37°C, fluorescence was determined using a Fluorescence/Multi-Detection Microplate Reader (Bio-Tek). Rotenone, 10 μmol/L, was used as a standard inhibitor of ΔΨm. Data (the ratio of aggregated and monomeric JC-1) were normalized to the control group and are expressed as a percentage of the control.

### Detection of Enzymatic Activity of Mitochondrial Oxidative Phosphorylation Complexes

Commercial assay kits (Solarbio, Beijing, China) were used to measure enzyme activity of mitochondrial oxidative phosphorylation (OXPHOS) complexes in BMSCs according to the manufacturer’s protocol. Complex I enzyme activity was determined by the change in absorbance of NADH at 340 nm. Complex II enzyme activity was determined by the change in absorbance of 2,6-dichlorophenol indophenol at 600 nm. Enzyme activity of complex III and complex IV was determined by the change in absorbance of reduced cytochrome c at 550 nm. Absorbance was determined using a Fluorescence/Multi-Detection Microplate Reader (Bio-Tek). Data were normalized to the control group and are expressed as a percentage of the control.

### Mito-Tracker Green Staining and Hoechst 33,342 Staining

Mito-tracker green staining and Hoechst 33,342 staining were used to label mitochondria and nuclei in BMSCs, respectively. At 72 h after transfection, cells were washed twice with phosphate buffered saline (PBS) and incubated with Mito-tracker green (Beyotime) for 30 min. Cells were then suspended in PBS, and 10 µL of Hoechst 33,342 dye was added (Beyotime). After washing twice with PBS, a fluorescence microscope (Nikon TE2000-U, Tokyo, Japan) was used to capture five randomly selected fields that were analyzed with NIS-Elements software.

### Oil Red O Staining and Quantification

Bone mesenchymal stem cells were seeded into 6-well culture plates. After transfection and differentiation for 5 days, differentiated BMSCs were washed with PBS and then fixed with 4% formaldehyde for 30 min. Differentiated BMSCs were dyed with oil red O working solution (BBI, Shanghai, China) for 60 min at room temperature and then washed three times with PBS, according to the manufacturer’s specification. After washing, a fluorescence inverted light microscope (Leica DMi8) was used to capture images. At the end, stain in cells was extracted by isopropanol and absorbance was measured at 510 nm with a Fluorescence/Multi-Detection Microplate Reader (Bio-Tek).

### Western Blot Analysis

Radio-immune precipitation assay buffer (Beyotime) with phenylmethane sulfonyl fluoride protease inhibitor (Beyotime) was used to lyse tissue and cellular proteins. The homogenate was centrifuged at 13,000 ×g for 10 min at 4°C. The supernatant was collected, and protein concentration was determined immediately using a bicinchoninic acid assay protein quantification kit (Beyotime). Proteins were separated in 10% sodium dodecyl sulfate–polyacrylamide gel electrophoresis, transferred onto a polyvinylidene difluoride membrane, and then probed with antibodies following standard procedures.

The following antibodies and their dilutions were used in western blot: mouse anti-PGC1 alpha antibody (1C1B2, 1:5,000; Proteintech, Rosemont, United States), rabbit anti-NRF1 antibody (AF7620, 1:1,000; Beyotime), rabbit anti-TOMM20 antibody (AF1717, 1:1,000; Beyotime), rabbit anti-PPAR gamma polyclonal antibody (bs-0530R, 1:1,000; Bioss, Beijing, China), rabbit anti-CEBP alpha polyclonal antibody (bs-24540R, 1:1,000; Bioss), rabbit anti-beta-actin antibody (bs-0061R, 1:5,000; Bioss), goat anti-rabbit IgG-HRP (BS13278, 1:10,000; Bioworld, Minnesota, United States), and goat anti-mouse IgG-HRP (BS12478, 1:10,000; Bioworld).

### Statistical Analyses

All experiments were performed at least three times. Data are presented as the mean ± standard error of the mean (SEM). Statistical analyses were performed using Student’s t-test, with statistical significance indicated as *p < 0.05, **p < 0.01, and ***p < 0.001.

## Results

### More Severe Fat Deposition in Sex-Linked Dwarf Chicken Bone Marrow Tissue

**FIGURE 1:**
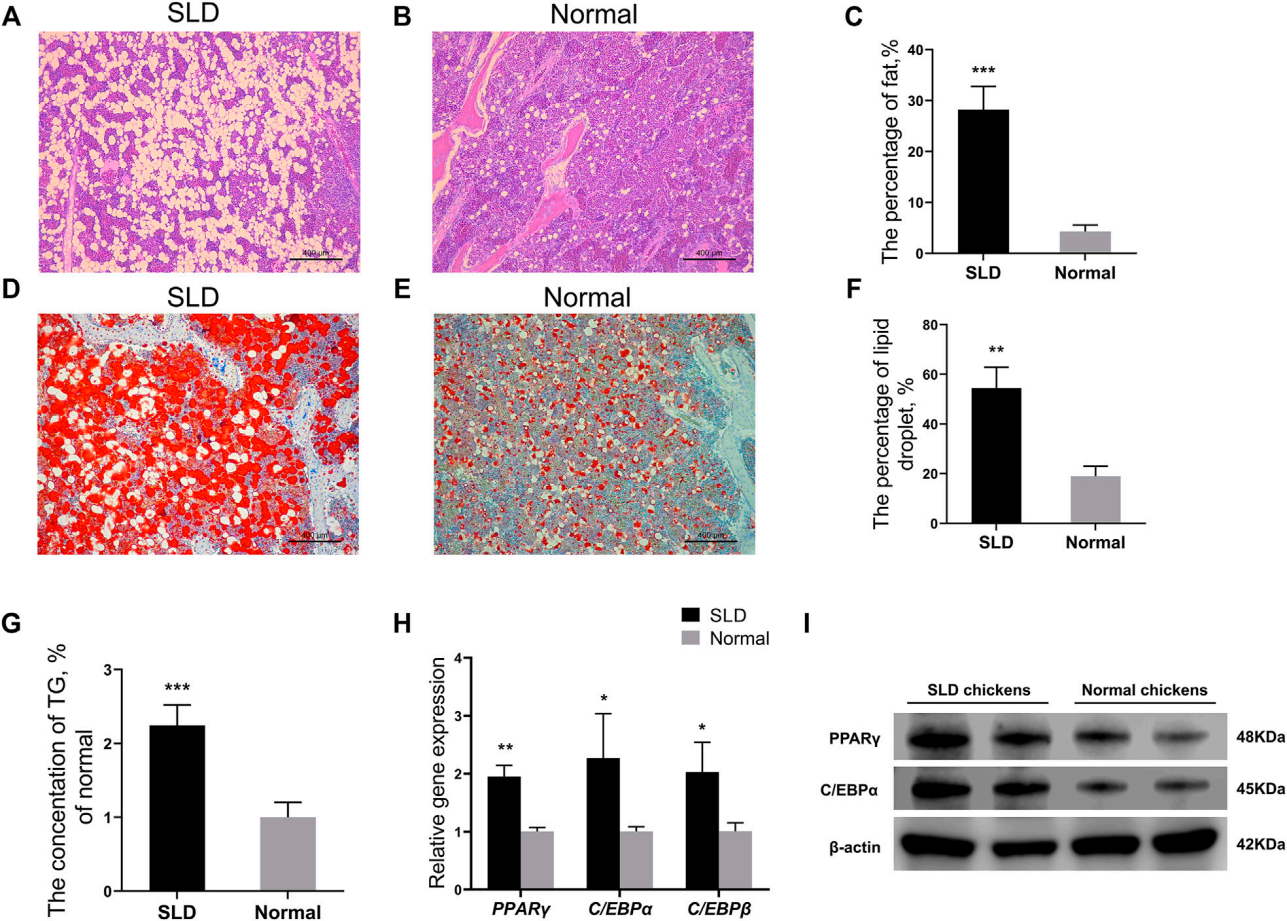
More severe fat deposition in SLD chicken bone marrow tissue. **(A,B)** Hematoxylin and eosin-stained paraffin sections of bone marrow tissue from SLD and normal chickens. Scale bar = 400 µm. **(C)** The percentage of fat in volume (%) in bone marrow tissue from SLD and normal chickens (*n* = 3). **(D,E)** Oil red O-stained frozen sections of bone marrow tissue from SLD and normal chickens. Scale bar = 400 µm. **(F)** The percentage of lipid droplets in volume (%) in bone marrow tissue from SLD and normal chickens (*n* = 3). **(G)** The triglyceride (TG) levels (% of normal) in bone marrow tissues of SLD and normal chickens (*n* = 3). **(H)** Expression of genes related to adipogenic differentiation in bone mesenchymal stem cells, as determined by reverse-transcription QPCR (*n* = 3). **(I)** Protein levels related to adipogenic differentiation in bone mesenchymal stem cells, as determined by western blot (*n* = 2). β-actin was used as the control. In all panels, data are presented as the mean ± S.E.M. of three biological replicates. *p < 0.05, **p < 0.01, ***p < 0.001.

To compare the differences in fat deposition of bone marrow tissues between SLD and normal chickens, we did pathological sections on bone marrow tissues of 21-day-old SLD and normal chickens. Hematoxylin and eosin staining of bone marrow tissues from SLD and normal chickens showed the percentage of fat in bone marrow tissues of SLD chickens was significantly higher than that of normal chickens (Figures 1A–C). Oil red O staining on bone marrow tissues of SLD and normal chickens showed the percentage of lipid droplets in bone marrow tissues of SLD chickens was higher than that of normal chickens (Figures 1D–F). Furthermore, triglyceride content in the bone marrow tissue of SLD chickens was highly significantly higher than that of normal chickens (Figure 1G). To investigate whether the cause of this phenotype in SLD chickens was due to differentiation of BMSCs, expression of marker genes of adipogenic differentiation in BMSCs of SLD and normal chickens was examined. Expression of the marker genes *PPARγ*, *C/EBPα*, and *C/EBPβ* increased significantly in SLD chickens (Figure 1H). The results were similar in protein level assays (Figure 1I). Thus, fat deposition in the bone marrow tissue of SLD chickens was much more severe than that of normal chickens. This result might be due to differentiation of BMSCs in SLD chickens.

### Mitochondrial Function and Mitochondrial Biogenesis Were Strengthened in Sex-Linked Dwarf Chicken Bone Mesenchymal Stem Cells

**FIGURE 2:**
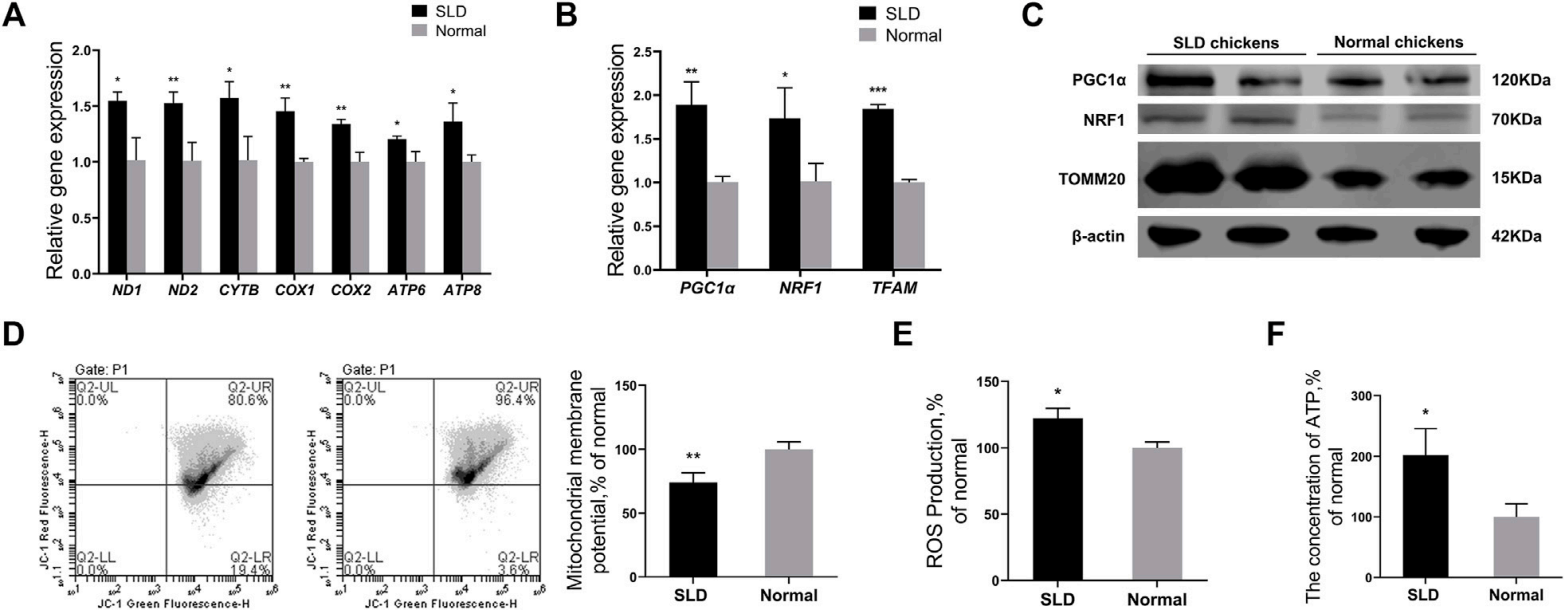
Mitochondrial function and mitochondrial biogenesis were strengthened in SLD chicken BMSCs. **(A)** Expression of genes related to mtDNA-encoded oxidative phosphorylation in bone mesenchymal stem cells (BMSCs), as determined by reverse-transcription (RT) qPCR (*n* = 3). **(B)** Expression of genes related to mitochondrial biogenesis in BMSCs, as determined by RT-qPCR (*n* = 3). **(C)** Protein levels of mitochondrial biogenesis and mitochondrial marker genes in BMSCs by western blot. β-actin was used as the control (*n* = 2). **(D)** Mitochondrial membrane potential in BMSCs (*n* = 3). Red fluorescence represents aggregation of JC-1, green fluorescence represents monomeric JC-1, ∆Ψm was represented as the ratio of aggregated and monomeric JC-1. **(E)** Reactive oxygen species (ROS) levels in BMSCs (*n* = 3). **(F)** The ATP levels in BMSCs (*n* = 3). In all panels, data are presented as the mean ± S.E.M. of three biological replicates. *p < 0.05, **p < 0.01, ***p < 0.001.

Mitochondria can synthesize ATP through oxidative phosphorylation to provide a major source of energy for adipogenic differentiation of MSCs (Zhang et al., 2013). Therefore, we speculated that mitochondria have an important role in the production of lipids in bone marrow tissue. We investigated the differences in mitochondrial function in the bone marrow tissue of SLD chickens and normal chickens. The mRNA levels of mtDNA-encoded OXPHOS-related and mitochondrial biogenesis-related genes in BMSCs of SLD and normal chickens were measured by RT-qPCR. Genes included *ND1*, *ND2*, *CYTB*, *COX1*, *COX2*, *ATP6*, *ATP8*, *PGC1α*, *NRF1*, and *TFAM*. Protein levels of PGC1α, NRF1, and TOMM20 in BMSCs of SLD and normal chickens were measured by western blot. The mRNA expression of genes was elevated significantly in BMSCs of SLD chickens (Figures 2A,B). Protein levels of PGC1α, NRF1, and TOMM20 also increased in SLD chickens (Figure 2C). Furthermore, to indicate mitochondrial function, ΔΨm, ROS production, and ATP content were examined. Compared with normal chickens, ΔΨm decreased and ROS production and ATP content increased in SLD chickens (Figure 2D–F **)**. Therefore, in SLD chickens, changes in mitochondrial function might affect adipogenic differentiation of BMSCs and thus adipogenesis.

### Growth Hormone Receptor Represses Mitochondrial Function and Mitochondrial Biogenesis in Chicken Bone Mesenchymal Stem Cells

**FIGURE 3:**
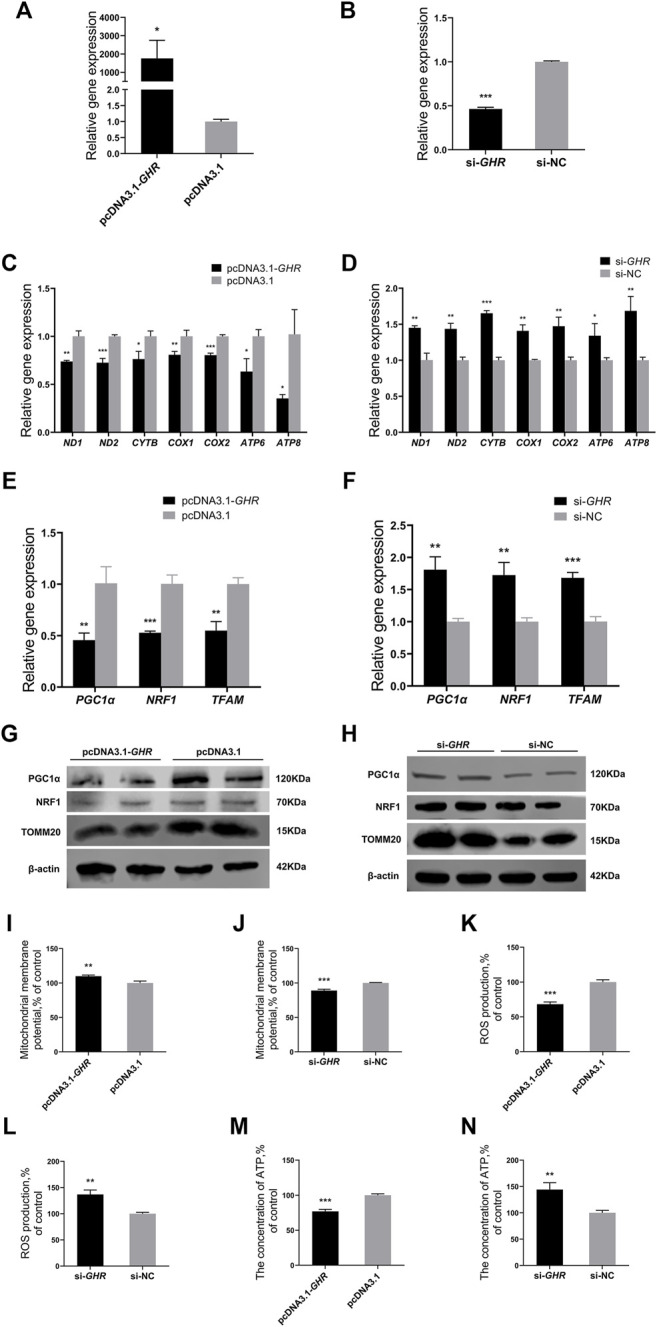
GHR represses mitochondrial function and mitochondrial biogenesis in chicken BMSCs. **(A,B)** Expression of GHR in BMSCs after being transfected with an overexpression vector or siRNA (*n* = 3). After overexpression and knockdown of GHR: **(C,D)** expression of genes related to mtDNA-encoded oxidative phosphorylation in BMSCs (*n* = 3); **(E,F)** expression of genes related to mitochondrial biogenesis in BMSCs (*n* = 3); **(G,H)** protein levels of PGC1α, NRF1, and TOMM20 (*n* = 2); **(I,J)** mitochondrial membrane potential in BMSCs (*n* = 3); **(K,L)** reactive oxygen species (ROS) production in BMSCs (*n* = 3); **(M,N)** the concentration of ATP in BMSCs (*n* = 3). In all panels, data are presented as the mean ± S.E.M. of three biological replicates. *p < 0.05, **p < 0.01, ***p < 0.001.

To investigate the effect of *GHR* on mitochondrial function, BMSCs were isolated and cultured *in vitro*, and then, *GHR* was overexpressed or knocked down. Efficiency of *GHR* overexpression and knockdown efficiency in chicken BMSCs differentiated for 72 h was examined by RT-qPCR. Compared with the control group, *GHR* expression was significantly upregulated after transfection with the vector of *GHR* (Figure 3A) and significantly downregulated after transfection with si-*GHR* (Figure 3B). Similar to *in vivo* experiments, mitochondrial function was examined after overexpression and knockdown of *GHR* in differentiated BMSCs. After overexpression of *GHR* in BMSCs, mRNA expression of mtDNA-encoded OXPHOS-related and mitochondrial biogenesis-related genes decreased (Figures 3C,E). Protein levels of PGC1α, NRF1, and TOMM20 showed similar results (Figure 3G). In addition, ΔΨm increased, and ROS production and ATP content decreased (Figures 3I, K, M). Opposite results were observed after knockdown of *GHR* (Figures 3D, F, H, J, L, N). These results suggested that *GHR* repressed mitochondrial function during adipogenic differentiation in chicken BMSCs.

### Growth Hormone Receptor Represses the Enzymatic Activity of Oxidative Phosphorylation Complexes in Chicken Bone Mesenchymal Stem Cells

**FIGURE 4:**
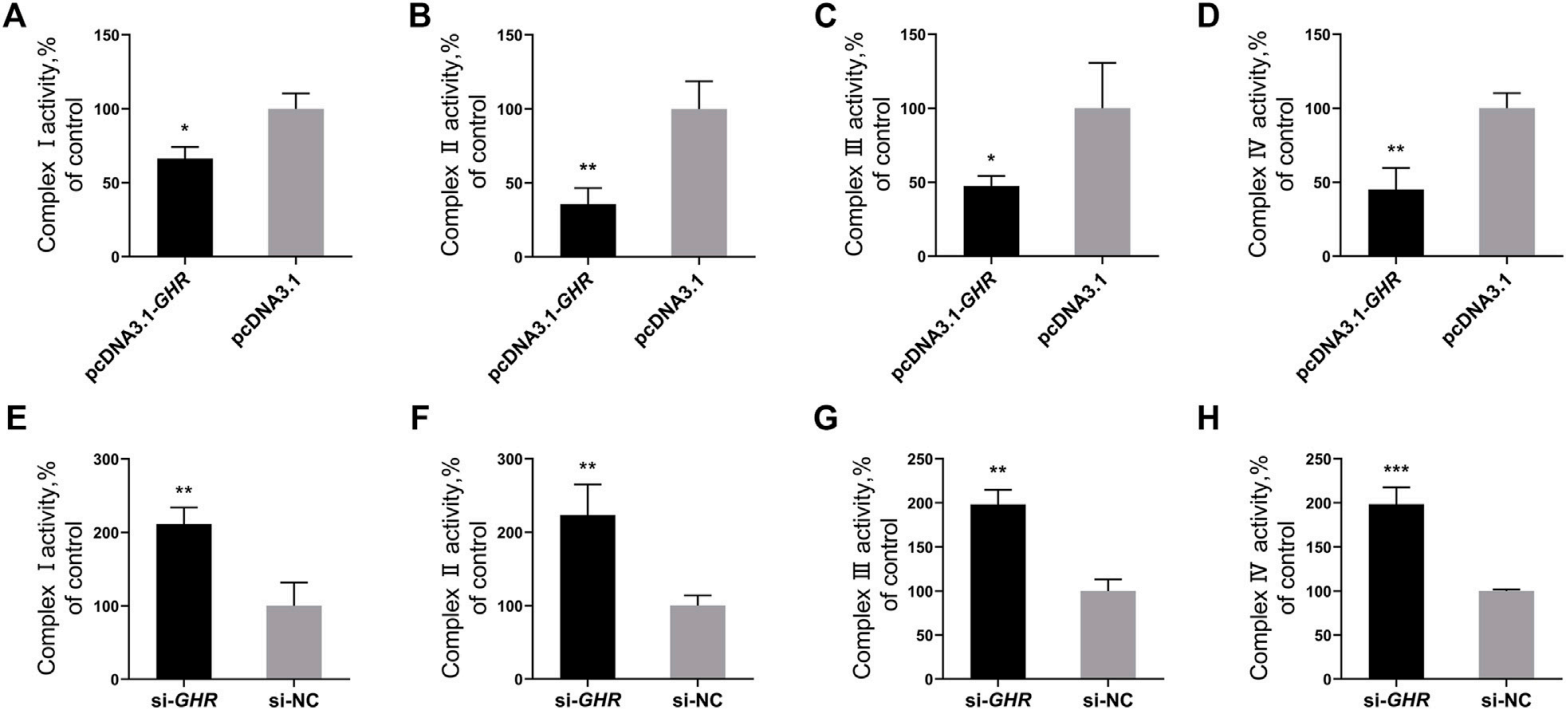
GHR represses the enzymatic activity of OXPHOS complexes in chicken BMSCs. After overexpression of GHR, enzymatic activity of complex I **(A)**, complex II **(B)**, complex III **(C)**, and complex IV **(D)** in bone mesenchymal stem cells. After knockdown of GHR, enzymatic activity of complex I **(E)**, complex II **(F)**, complex III **(G)**, and complex IV **(H)** in bone mesenchymal stem cells. In all panels, data are presented as the mean ± S.E.M. of three biological replicates. *p < 0.05, **p < 0.01, ***p < 0.001.

To further investigate the effects of *GHR* on mitochondrial function in chicken BMSCs during adipogenic differentiation, enzyme activity of mitochondrial OXPHOS complexes was examined in differentiated BMSCs after overexpression and knockdown of *GHR*. Enzymatic activities of OXPHOS complexes I, II, III, and IV decreased significantly in chicken BMSCs after overexpression of *GHR* (Figures 4A–G), whereas after *GHR* knockdown, enzymatic activities increased significantly (Figures 4E–H). Overall, the results indicated that *GHR* repressed mitochondrial function by suppressing enzymatic activity of OXPHOS complexes in chicken BMSCs during adipogenic differentiation.

### Growth Hormone Receptor Reduces Mitochondrial Number by Mitochondrial Biogenesis in Chicken Bone Mesenchymal Stem Cells

**FIGURE 5:**
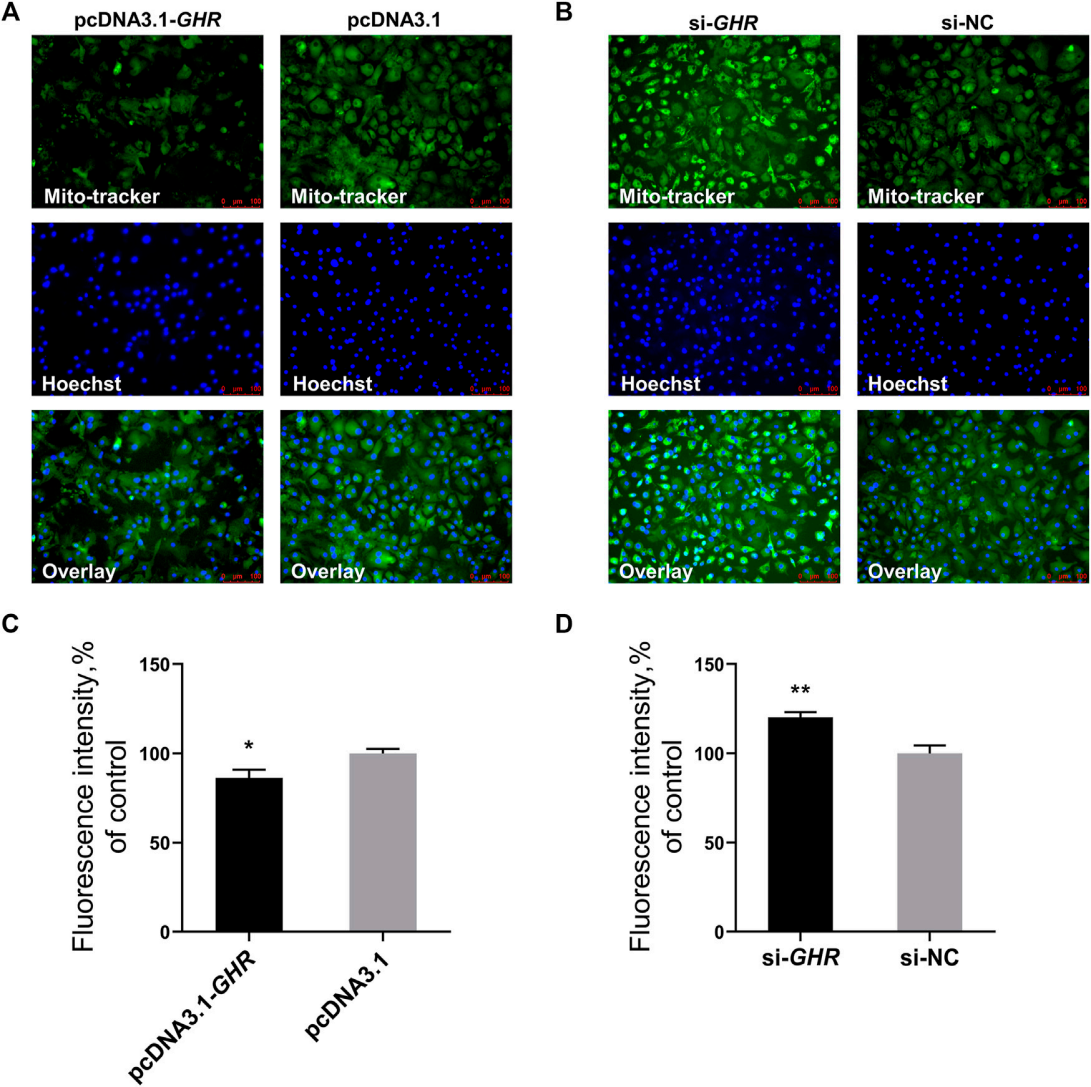
GHR reduces mitochondrial number by mitochondrial biogenesis in chicken BMSCs. After overexpression and knockdown of GHR: **(A,B)** mito-tracker staining of differentiated BMSCs; **(C,D)** fluorescence intensity of Mito-tracker staining (*n* = 3). In all panels, data are presented as the mean ± S.E.M. of three biological replicates. *p < 0.05, **p < 0.01.

The previous results showed that *GHR* suppressed mRNA and protein levels of critical genes for mitochondrial biogenesis, and we further explored the effects of *GHR* on mitochondrial biogenesis in chicken BMSCs. Mitochondrial biogenesis increases the number of mitochondria to meet intracellular energy requirements. Therefore, the effect of *GHR* on mitochondrial function was explored by assaying the number of mitochondria. Mito-tracker staining was used to label mitochondria, with fluorescence intensity representing mitochondrial quantity. After overexpression of *GHR* in BMSCs, fluorescence intensity weakened, and the number of mitochondria decreased (Figures 5A, C). After knockdown of *GHR*, fluorescence intensity strengthened, and the number of mitochondria increased (Figures 5B, D). Thus, in addition to repressing mitochondrial function, *GHR* reduced mitochondrial number and quality by repressing mitochondrial biogenesis during adipogenic differentiation of chicken BMSCs.

### Growth Hormone Receptor Represses Chicken Bone Mesenchymal Stem Cells’ Adipogenic Differentiation

**FIGURE 6:**
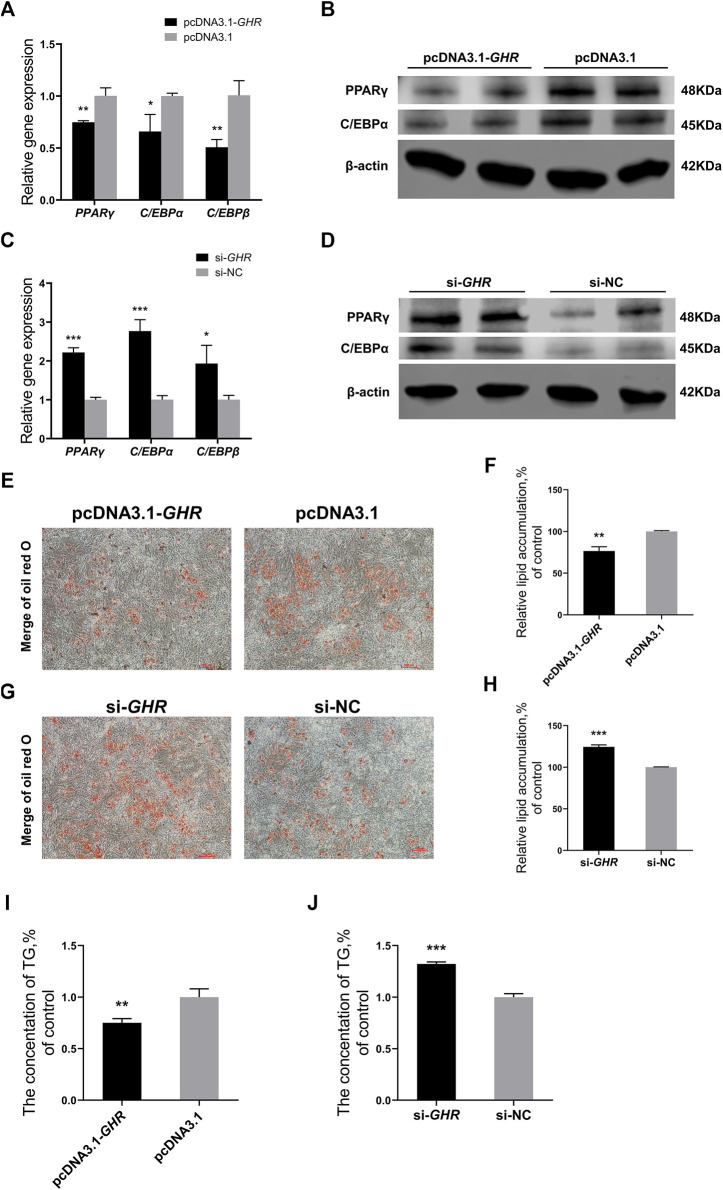
GHR represses chicken BMSCs’ adipogenic differentiation. **(A,B)** Expression of genes (*n* = 3) and protein levels (*n* = 2) associated with adipogenic differentiation after overexpression of GHR. **(C,D)** Expression of genes (*n* = 3) and protein levels (*n* = 2) associated with adipogenic differentiation after knockdown of GHR. **(E,F)** Oil red O test and lipid droplet quantification in BMSCs differentiated for 5 days after overexpression of GHR (*n* = 3). Scale bars = 100 µm. **(G,H)** Oil red O test and lipid droplet quantification in BMSCs differentiated for 5 days after knockdown of GHR (*n* = 3). Scale bars = 100 µm. **(I,J)** The triglyceride (TG) contents in BMSCs after overexpression and knockdown of GHR (*n* = 3). In all panels, data are presented as the mean ± S.E.M. of three biological replicates. *p < 0.05, **p < 0.01, ***p < 0.001.

Finally, *GHR* was overexpressed and knocked down in chicken BMSCs to investigate the effects of *GHR* on adipogenic differentiation. Adipogenic differentiation was induced in BMSCs, and expression of associated genes was detected by RT-qPCR. The genes were *PPARγ*, *C/EBPα*, and *C/EBPβ*. Protein levels of PPARγ and C/EBPα in chicken BMSCs differentiated for 72 h were measured simultaneously by western blot. Expression of adipogenic differentiation-related genes was significantly downregulated after overexpression of *GHR* (Figure 6A), whereas after knockdown of *GHR*, expression was significantly upregulated (Figure 6C). Protein levels of PPARγ and C/EBPα in chicken BMSCs showed similar results (Figures 6B,D). Furthermore, the oil red O test was used to measure lipid droplet content in chicken BMSCs differentiated for 5 days after overexpression and knockdown of *GHR*. Overexpression of *GHR* depressed the lipid droplet depot in BMSCs, whereas knockdown had the opposite effect (Figures 6E–H). In addition, overexpression of *GHR* repressed triglyceride production in BMSCs (Figure 6I), whereas knockdown of *GHR* (Figure 6J) produced opposite results. Thus, *GHR* repressed fat deposition in chickens by inhibiting adipogenic differentiation of chicken BMSCs.

## Discussion

Since their discovery in 1940, research on SLD chickens has been uninterrupted. Mutation of the *GHR* gene in SLD chickens interferes with binding of GH to GHR (Lin et al., 2012), and therefore, SLD chickens are a specific animal model for mutation of the *GHR* gene (Agarwal et al., 1995). Fat deposition is more severe in SLD chickens than in normal chickens (Guillaume, 1976). In a previous study, compared with normal chickens, red bone marrow was severely depleted and replaced by yellow bone marrow in 7-week-old SLD chickens (Li et al., 2021). It was hypothesized that the SLD phenotype was due to a functional deletion of the *GHR* gene. Therefore, in this study, the relation between *GHR* and adipogenic differentiation of BMSCs was explored.

In this study, fat deposition in bone marrow tissue of 21-day-old SLD chickens was greater than that in normal chickens, consistent with previous findings (Li et al., 2021). In addition, triglyceride content of bone marrow tissue in SLD chickens was twice as high as that in normal chickens, consistent with fat deposition. Fat in bone marrow tissue is primarily derived from adipogenic differentiation of MSCs (Hardouin et al., 2014). The balance between adipose and bone tissues in bone marrow tissue is maintained primarily by two types of MSC differentiation: adipogenic and osteogenic (Duque, 2008). When adipogenic differentiation of MSCs increases, osteogenic differentiation is relatively weakened, resulting in fat deposition (Nuttall et al., 2014). Therefore, it was hypothesized that the more severe fat deposition in SLD bone marrow tissue was due to increased adipogenic differentiation capacity of BMSCs in SLD chickens because of deficiency in normal *GHR* gene function. To test the hypothesis, BMSCs were extracted from SLD and normal chickens, and differences in expression of genes associated with adipogenic differentiation in the two groups of cells were examined. Expression of *PPARγ*, *C/EBPα*, and *C/EBPβ* in BMSCs was significantly higher in SLD chickens than in normal chickens. *PPARγ*, as the predominant transcription factor in adipocyte differentiation, also plays an important role in adipogenic differentiation of BMSCs. In one study, addition of the *PPARγ* agonist rosiglitazone activated adipogenic differentiation of mouse BMSCs (Gimble et al., 1996). During adipogenic differentiation of MSCs, *C/EBPβ*, *PPARγ*, and *C/EBPα* are sequentially activated (Zhao et al., 2015).

An increasing number of studies show that regulation of mitochondrial dynamics and function is critical for successful differentiation of MSCs. Adipogenic differentiation of MSCs is accompanied by changes in the mitochondrial phenotype, including increased mitochondrial biogenesis and abundance of OXPHOS complexes (Hofmann et al., 2012). Therefore, mitochondrial function of BMSCs from SLD and normal chickens was examined by using ATP content, ROS, and ΔΨm assays. The ATP content was higher in the BMSCs of SLD chickens, indicating that deficiency in *GHR* function led to an increase in mitochondrial oxidative phosphorylation capacity and therefore production of ATP. Those changes provided the necessary conditions for adipogenic differentiation of BMSCs. Reactive oxygen species are produced by the OXPHOS pathway associated with energy production in mitochondria (Madamanchi and Runge, 2007). Only unregulated levels of ROS are harmful, whereas regulated ROS production is needed for essential signaling pathways that regulate cell functions (Guzik and Harrison, 2006). Production of ROS by mitochondrial complex III is required to activate adipogenic differentiation of MSCs, and ROS levels increase during adipogenesis induction in MSCs (Tormos et al., 2011). In this study, ROS production was greater in BMSCs of SLD chickens than in those of normal chickens. Reactive oxygen species promote lipid accumulation in human adipose stromal cells undergoing adipogenesis (Higuchi et al., 2013). Therefore, increases in ROS may be one factor that stimulates differentiation of BMSCs toward adipogenesis. Notably, ΔΨm decreased in BMSCs of SLD chickens compared with that in normal chickens. In general, the higher ΔΨm is, the greater the energy capacity of the inner mitochondrial membrane and the higher the amount of ATP synthesis (Zorova et al., 2018). However, maintaining excessively high or excessively low ΔΨm can be harmful to mitochondria and cells (Skulachev, 1996; Rajasekaran et al., 2007). A decrease in ΔΨm is presumed to be due to the effect of “mild uncoupling of mitochondria”, which ensures the supply of ATP while appropriately lowering the ΔΨm in response to damage caused by elevated ROS(M. Cunha et al., 2011). In addition, expression of mitochondrial genes encoding OXPHOS, including *ND1*, *ND2*, *CYTB*, *COX1*, *COX2*, *ATP6,* and *ATP8*, and genes related to mitochondrial biogenesis, including *PGC1α*, *NRF1*, and *TFAM*, were upregulated in BMSCs of SLD chickens compared with those in normal chickens. Levels of the mitochondrial membrane protein TOMM20 and mitochondrial biogenesis proteins (PGC1α, NRF1) also increased. The results indicated that increases in mitochondrial function in BMSCs of SLD chickens were due to the absence of *GHR* function. Therefore, changes in mitochondria in BMSCs of SLD chickens may affect adipogenic differentiation of BMSCs and ultimately increase fat deposition in SLD chickens.

To further confirm the conjecture on the mechanism of fat deposition in bone marrow tissue of SLD chickens, BMSCs were extracted from chickens for cellular verification. Expression of mitochondrial genes encoding OXPHOS and genes associated with mitochondrial biogenesis was examined after overexpression and knockdown of *GHR* in BMSCs. The results were consistent with those obtained *in vivo*. When *GHR* was knocked down in chicken BMSCs, ΔΨm decreased, ROS production and ATP content increased, and protein levels of PGC1α, NRF1, and TOMM20 was enhanced. The opposite result after overexpression of *GHR*. Collectively, the results suggest that *GHR* inhibits mitochondrial function and mitochondrial biogenesis during adipogenic differentiation in chicken BMSCs. This conclusion is also supported by enzymatic activity of complexes I, II, III, and IV after overexpression and knockdown of *GHR*. Enzymatic activity of complexes I, II, III, and IV was enhanced after knockdown of *GHR*. The opposite result after overexpression of *GHR*. Complexes I, II, III, and IV are important components of the mitochondrial electron transport chain and are involved in the adipogenic differentiation of BMSCs through mitochondrial oxidative phosphorylation. In one study, inhibition of the mitochondrial electron transport chain suppressed adipogenic differentiation of MSCs (Zhang et al., 2013). Mitochondrial biogenesis is regulation of the number of mitochondria through mitochondrial self-renewal in response to energy demands triggered by developmental signals and environmental stressors (Popov, 2020). Mitochondrial biogenesis increases during adipogenic differentiation of MSCs (Zhang et al., 2013). In immortalized human MSCs, overexpression of *PGC-1α* increases mitochondrial function and biogenesis and promotes adipogenic differentiation of MSCs (Hao et al., 2020). *TFAM* can bind to the mitochondrial light strand promoter and functions in mitochondrial transcription regulation (Hao et al., 2020), and knockdown of *TFAM* in MSCs inhibits adipogenic differentiation (Zhang et al., 2013). Furthermore, Mito-tracker staining validated the effect of *GHR* on mitochondrial biogenesis. The number of mitochondria decreased after overexpression of *GHR*, indicating that *GHR* inhibited mitochondrial biogenesis. The opposite result was observed after knockdown of *GHR*.

In addition, whether *GHR* inhibited adipogenic differentiation of chicken BMSCs *in vitro* was investigated. After overexpression of *GHR* in chicken BMSCs, expression of differentiation-related genes, including *PPARγ*, *C/EBPα*, and *C/EBPβ*, was repressed and lipid droplet production and triglyceride levels decreased. Notably, *PPARγ* determines the direction of adipogenic differentiation of MSCs (Zhuang et al., 2016). With knockdown of *GHR*, opposite results were obtained. Thus, *GHR* can inhibit adipogenic differentiation of chicken BMSCs.

In conclusion, sex-linked dwarf chickens had severe fat deposition in bone marrow tissue than normal chickens. Increased adipogenic differentiation of BMSCs in SLD chickens was associated with increases in mitochondrial biogenesis and function and expression of genes related to differentiation. After overexpression of *GHR* in chicken BMSCs, mitochondrial function, mitochondrial biogenesis and adipogenic differentiation of BMSCs were repressed. The opposite results were observed after knockdown of *GHR*. Therefore, *GHR* inhibits excessive adipogenic differentiation of chicken BMSCs by repressing mitochondrial biogenesis and mitochondrial function. This suppression might explain the clinical manifestation of severe fat deposition in SLD chickens.

## Data Availability

The original contributions presented in the study are included in the article/Supplementary Material, further inquiries can be directed to the corresponding authors.

## References

[B1] Agarwal S. K. Cogburn L. A. Burnside J. (1995). Comparison of Gene Expression in Normal and Growth Hormone Receptor-Deficient Dwarf Chickens Reveals a Novel Growth Hormone-Regulated Gene. Biochem. Biophysical Res. Commun. 206, 153–160. 10.1006/bbrc.1995.1022 7818515

[B2] Attia N. Mashal M. (2020). Mesenchymal Stem Cells: The Past Present and Future. Adv. Exp. Med. Biol. 1312, 107–129. 10.1007/5584_2020_595 33159306

[B3] Brown-Borg H. M. Rakoczy S. G. Sharma S. Bartke A. (2009). Long-living Growth Hormone Receptor Knockout Mice: Potential Mechanisms of Altered Stress Resistance. Exp. Gerontol. 44, 10–19. 10.1016/j.exger.2008.07.002 18675334 PMC2743895

[B4] Burnside J. Liou S. S. Cogburn L. A. Burnside J. (1991). Molecular Cloning of the Chicken Growth Hormone Receptor Complementary Deoxyribonucleic Acid: Mutation of the Gene in Sex-Linked Dwarf Chickens*. Endocrinology 128, 3183–3192. 10.1210/endo-128-6-3183 2036984

[B5] Dehkhoda F. Lee C. M. M. Medina J. Brooks A. J. (2018). The Growth Hormone Receptor: Mechanism of Receptor Activation, Cell Signaling, and Physiological Aspects. Front. Endocrinol. 9, 1–23. 10.3389/fendo.2018.00035 PMC5816795 29487568

[B6] Duque G. (2008). Bone and Fat Connection in Aging Bone. Curr. Opin. Rheumatol. 20, 429–434. 10.1097/BOR.0b013e3283025e9c 18525356

[B7] Gesing A. Bartke A. Wang F. Karbownik-Lewinska M. Masternak M. M. (2011a). Key Regulators of Mitochondrial Biogenesis Are Increased in Kidneys of Growth Hormone Receptor Knockout (GHRKO) Mice. Cell Biochem. Funct. 29, 459–467. 10.1002/cbf.1773 21755522 PMC3682479

[B8] Gesing A. Masternak M. M. Wang F. Joseph A.-M. Leeuwenburgh C. Westbrook R. (2011b). Expression of Key Regulators of Mitochondrial Biogenesis in Growth Hormone Receptor Knockout (GHRKO) Mice Is Enhanced but Is Not Further Improved by Other Potential Life-Extending Interventions. Journals Gerontol. Ser. A: Biol. Sci. Med. Sci. 66A, 1062–1076. 10.1093/gerona/glr080 PMC3172561 21788651

[B9] Gimble J. M. Robinson C. E. Wu X. Kelly K. A. Rodriguez B. R. Kliewer S. A. (1996). Peroxisome Proliferator-Activated Receptor-Gamma Activation by Thiazolidinediones Induces Adipogenesis in Bone Marrow Stromal Cells. Mol. Pharmacol. 50, 1087–1094. 8913339

[B10] Guillaume J. (1976). The Dwarfing Gene Dw: Its Effects on Anatomy, Physiology, Nutrition, Management. Its Application in Poultry Industry. World's Poult. Sci. J. 32, 285–305. 10.1079/wps19760009

[B11] Guzik T. J. Harrison D. G. (2006). Vascular NADPH Oxidases as Drug Targets for Novel Antioxidant Strategies. Drug Discov. Today 11, 524–533. 10.1016/j.drudis.2006.04.003 16713904

[B12] Hao Z. Wu T. Cui X. Zhu P. Tan C. Dou X. (2020). N6-Deoxyadenosine Methylation in Mammalian Mitochondrial DNA. Mol. Cel 78, 382–395. 10.1016/j.molcel.2020.02.018 PMC7214128 32183942

[B13] Hardouin P. Pansini V. Cortet B. (2014). Bone Marrow Fat. Jt. Bone Spine 81, 313–319. 10.1016/j.jbspin.2014.02.013 24703396

[B14] Higuchi M. Dusting G. J. Peshavariya H. Jiang F. Hsiao S. T.-F. Chan E. C. (2013). Differentiation of Human Adipose-Derived Stem Cells into Fat Involves Reactive Oxygen Species and Forkhead Box O1 Mediated Upregulation of Antioxidant Enzymes. Stem Cell Dev. 22, 878–888. 10.1089/scd.2012.0306 PMC3585477 23025577

[B15] Hofmann A. D. Beyer M. Krause-Buchholz U. Wobus M. Bornhäuser M. Rödel G. (2012). OXPHOS Supercomplexes as a Hallmark of the Mitochondrial Phenotype of Adipogenic Differentiated Human MSCs. PLoS One 7, e35160. 10.1371/journal.pone.0035160 22523573 PMC3327658

[B16] Hsu Y.-C. Wu Y.-T. Yu T.-H. Wei Y.-H. (2016). Mitochondria in Mesenchymal Stem Cell Biology and Cell Therapy: From Cellular Differentiation to Mitochondrial Transfer. Semin. Cel Dev. Biol. 52, 119–131. 10.1016/j.semcdb.2016.02.011 26868759

[B17] Hu B. Hu S. Yang M. Liao Z. Zhang D. Luo Q. (2019). Growth Hormone Receptor Gene Is Essential for Chicken Mitochondrial Function *In Vivo* and *In Vitro* . Ijms 20, 1608–1613. 10.3390/ijms20071608 PMC6480491 30935132

[B18] Hu B. Li H. Zhang X. (2021). A Balanced Act: The Effects of GH-GHR-IGF1 Axis on Mitochondrial Function. Front. Cel Dev. Biol. 9, 1–14. 10.3389/fcell.2021.630248 PMC8012549 33816476

[B19] Huang P.-I. Chen Y.-C. Chen L.-H. Juan C.-C. Ku H.-H. Wang S.-T. (2011). PGC-1α Mediates Differentiation of Mesenchymal Stem Cells to Brown Adipose Cells. Jat 18, 966–980. 10.5551/jat.7401 21817823

[B20] James A. W. (20132013). Review of Signaling Pathways Governing MSC Osteogenic and Adipogenic Differentiation. Scientifica 2013, 1–17. 10.1155/2013/684736 PMC3874981 24416618

[B21] Li H. Hu B. Hu S. Luo W. Sun D. Yang M. (2021). High Expression of BCL6 Inhibits the Differentiation and Development of Hematopoietic Stem Cells and Affects the Growth and Development of Chickens. J. Anim. Sci. Biotechnol. 12, 18–13. 10.1186/s40104-020-00541-3 33541426 PMC7863511

[B22] Li H. Hu B. Luo Q. Hu S. Luo Y. Zhao B. (2019). Runting and Stunting Syndrome Is Associated with Mitochondrial Dysfunction in Sex-Linked Dwarf Chicken. Front. Genet. 10, 1–11. 10.3389/fgene.2019.01337 32010193 PMC6978286

[B23] Li Y. Jin D. Xie W. Wen L. Chen W. Xu J. (2018). PPAR-γ and Wnt Regulate the Differentiation of MSCs into Adipocytes and Osteoblasts Respectively. Cscr 13, 185–192. 10.2174/1574888x12666171012141908 29034841

[B24] Lin S. Li H. Mu H. Luo W. Li Y. Jia X. (2012). Let-7b Regulates the Expression of the Growth Hormone Receptor Gene in Deletion-type dwarf Chickens. BMC Genomics 13, 306. 10.1186/1471-2164-13-306 22781587 PMC3428657

[B25] Liu Z. Solesio M. E. Schaffler M. B. Frikha‐Benayed D. Rosen C. J. Werner H. (2019). Mitochondrial Function Is Compromised in Cortical Bone Osteocytes of Long‐Lived Growth Hormone Receptor Null Mice. J. Bone Miner. Res. 34, 106–122. 10.1002/jbmr.3573 30216544 PMC7080402

[B26] Madamanchi N. R. Runge M. S. (2007). Mitochondrial Dysfunction in Atherosclerosis. Circ. Res. 100, 460–473. 10.1161/01.RES.0000258450.44413.96 17332437

[B27] M. Cunha F. M. Cerqueira F. J. Kowaltowski A. F. Kowaltowski J. (2011). Mild Mitochondrial Uncoupling as a Therapeutic Strategy. Cdt 12, 783–789. 10.2174/138945011795528778 21275885

[B28] Muruganandan S. Roman A. A. Sinal C. J. (2009). Adipocyte Differentiation of Bone Marrow-Derived Mesenchymal Stem Cells: Cross Talk with the Osteoblastogenic Program. Cell. Mol. Life Sci. 66, 236–253. 10.1007/s00018-008-8429-z 18854943 PMC11131547

[B29] Nuttall M. E. Shah F. Singh V. Thomas-Porch C. Frazier T. Gimble J. M. (2014). Adipocytes and the Regulation of Bone Remodeling: A Balancing Act. Calcif. Tissue Int. 94, 78–87. 10.1007/s00223-013-9807-6 24101233

[B30] Olarescu N. C. Berryman D. E. Householder L. A. Lubbers E. R. List E. O. Benencia F. (2015). GH Action Influences Adipogenesis of Mouse Adipose Tissue-Derived Mesenchymal Stem Cells. J. Endocrinol. 226, 13–23. 10.1530/JOE-15-0012 25943560 PMC4560118

[B31] Perret-Vivancos C. Abbate A. Ardail D. Raccurt M. Usson Y. Lobie P. E. (2006). Growth Hormone Activity in Mitochondria Depends on GH Receptor Box 1 and Involves Caveolar Pathway Targeting. Exp. Cel Res. 312, 215–232. 10.1016/j.yexcr.2005.10.027 16352305

[B32] Ploumi C. Daskalaki I. Tavernarakis N. (2017). Mitochondrial Biogenesis and Clearance: a Balancing Act. FEBS J. 284, 183–195. 10.1111/febs.13820 27462821

[B33] Popov L. D. (2020). Mitochondrial Biogenesis: An Update. J. Cell. Mol. Medi 24, 4892–4899. 10.1111/jcmm.15194 PMC7205802 32279443

[B34] Rajasekaran N. S. Connell P. Christians E. S. Yan L.-J. Taylor R. P. Orosz A. (2007). Human αB-Crystallin Mutation Causes Oxido-Reductive Stress and Protein Aggregation Cardiomyopathy in Mice. Cell 130, 427–439. 10.1016/j.cell.2007.06.044 17693254 PMC2962423

[B35] Skulachev V. P. (1996). Role of Uncoupled and Non-coupled Oxidations in Maintenance of Safely Low Levels of Oxygen and its One-Electron Reductants. Quart. Rev. Biophys. 29, 169–202. 10.1017/s0033583500005795 8870073

[B36] Tormos K. V. Anso E. Hamanaka R. B. Eisenbart J. Joseph J. Kalyanaraman B. (2011). Mitochondrial Complex III ROS Regulate Adipocyte Differentiation. Cel Metab. 14, 537–544. 10.1016/j.cmet.2011.08.007 PMC3190168 21982713

[B37] Wang X. Hai C. (2015). Redox Modulation of Adipocyte Differentiation: Hypothesis of “Redox Chain” and Novel Insights into Intervention of Adipogenesis and Obesity. Free Radic. Biol. Med. 89, 99–125. 10.1016/j.freeradbiomed.2015.07.012 26187871

[B38] Yang F. Yuanwei P.-w. Hao, Y. Q. Hao Y.-Q. Lu Z.-M. (2014). Emodin Enhances Osteogenesis and Inhibits Adipogenesis. BMC Complement. Altern. Med. 14.73 10.1186/1472-6882-14-74 24565373 PMC3974048

[B39] Zhang Y. Marsboom G. Toth P. T. Rehman J. (2013). Mitochondrial Respiration Regulates Adipogenic Differentiation of Human Mesenchymal Stem Cells. PLoS One 8, e77077. 10.1371/journal.pone.0077077 24204740 PMC3800007

[B40] Zhao X.-Y. Chen X.-Y. Zhang Z.-J. Kang Y. Liao W.-M. Yu W.-H. (2015). Expression Patterns of Transcription Factor PPARγ and C/EBP Family Members during *In Vitro* Adipogenesis of Human Bone Marrow Mesenchymal Stem Cells. Cell Biol. Int. 39, 457–465. 10.1002/cbin.10415 25523390

[B41] Zhuang H. Zhang X. Zhu C. Tang X. Yu F. Wei Shang G. (2016). Molecular Mechanisms of PPAR-γ; Governing MSC Osteogenic and Adipogenic Differentiation. Cscr 11, 255–264. 10.2174/1574888x10666150531173309 26027680

[B42] Zorova L. D. Popkov V. A. Plotnikov E. Y. Silachev D. N. Pevzner I. B. Jankauskas S. S. (2018). Mitochondrial Membrane Potential. Anal. Biochem. 552, 50–59. 10.1016/j.ab.2017.07.009 28711444 PMC5792320

